# Canid alphaherpesvirus 1 infection alters the gene expression and secretome profile of canine adipose-derived mesenchymal stem cells in vitro

**DOI:** 10.1186/s12985-024-02603-8

**Published:** 2024-12-27

**Authors:** Marina Prišlin Šimac, Šimun Naletilić, Vjekoslava Kostanić, Valentina Kunić, Tomaž Mark Zorec, Mario Poljak, Doroteja Vlaj, Rok Kogoj, Nenad Turk, Dragan Brnić

**Affiliations:** 1 Virology Department, Croatian Veterinary Institute, Zagreb, Croatia; 2 Department for Pathological Morphology, Croatian Veterinary Institute, Zagreb, Croatia; 3 Institute of Microbiology and Immunology, Faculty of Medicine, University of Ljubljana, Ljubljana, Slovenia; 4 Department of Microbiology and Infectious Diseases With Clinic, Faculty of Veterinary Medicine, University of Zagreb, Zagreb, Croatia

**Keywords:** Mesenchymal stem cell, Canine stem cells, In vitro, Gene expression, Secretome, Canine herpesvirus, Virus infection, Veterinary regenerative medicine

## Abstract

**Background:**

Canine adipose-derived mesenchymal stem cells (cAD-MSCs) demonstrate promising tissue repair and regeneration capabilities. However, the procurement and preservation of these cells or their secreted factors for therapeutic applications pose a risk of viral contamination, and the consequences for cAD-MSCs remain unexplored. Consequently, this research sought to assess the impact of canid alphaherpesvirus 1 (CHV) on the functional attributes of cAD-MSCs, including gene expression profiles and secretome composition.

**Methods:**

To this end, abdominal adipose tissue from 12 healthy dogs was harvested to isolate cAD-MSCs. These samples were tested for CHV contamination before introducing a wild-type CHV strain via serial passages. Following CHV infection, real-time reverse transcription-polymerase chain reaction array and liquid chromatography with tandem mass spectrometry assessments enabled analyses of gene expression and secretome’s proteomic profile, respectively.

**Results:**

This study showed that the initial cAD-MSC populations were devoid of CHV. cAD-MSCs showed susceptibility to infection with wild-type CHV, leading to notable modifications in gene expression and secretome profile. The observed genomic variations in gene expression indicate potential impacts on the stemness, migration, and other functional properties of cAD-MSCs, highlighting the need for further studies to evaluate their functional capacity post-infection. Moreover, gene expression and secretome analyses suggest a shift in stem cell differentiation toward an adipogenic phenotype.

**Conclusion:**

To the best of our knowledge, this is the first study of the effects of virus infection on gene expression and secretome composition in cAD-MSCs. The outcomes of our study underscore the imperative of routine viral screening prior to the therapeutic use of cAD-MSCs. Moreover, these findings provide novel insights into the pathogenic mechanisms of CHV and pave the way for future canine stem cell and virus research.

**Supplementary Information:**

The online version contains supplementary material available at 10.1186/s12985-024-02603-8.

## Background

Mesenchymal stem cells (MSCs) have the capacity to regenerate tissue in various species, including canines, presenting significant potential for treating diseases with limited therapeutic options, such as osteoarthritis, spinal cord injuries, and chronic skin wounds [1–3]. One promising stem cell source in canines is adipose tissue, abundant with canine adipose-derived mesenchymal stem cells (cAD-MSCs), which are easy to obtain and proliferate rapidly compared to other types of MSCs [4–6]. The cAD-MSCs exhibit notable regenerative properties, particularly the ability to modulate immune responses by secreting relevant molecules, i.e., the secretome [5, 7, 8]. Moreover, the application of the cAD-MSC secretome for therapy, rather than the cells themselves, offers the potential for numerous lower-risk treatments [9–12].

Acquiring and storing cAD-MSCs and secretomes in therapeutic quantities necessitates in vitro cultivation, thereby introducing the risk of microbial contamination. Studies have indicated that stored MSC batches may be contaminated with bacteria, fungi, or viruses [13, 14]. Bacterial and fungal contamination can be effectively mitigated with antibiotics and antimycotics [13]; however, mitigating potential viral contamination remains challenging. Available data indicate that MSCs can be permissive to infection with RNA and DNA viruses, which can lead to cell death, persistent infection, or cellular transformation and may ultimately impair their functionality [15–19]. Viral infection of MSCs has been associated with the inhibition of differentiation [20], increased secretion of proinflammatory cytokines [21], and loss of immunomodulatory function [22]. In cAD-MSCs, one study reported the susceptibility to distemper virus [23], and recent findings confirmed the possibility of viral contamination of cryobanked cAD-MSC batches by detecting canine parvovirus, influenza, parainfluenza, and canid alphaherpesvirus 1 [24].

Canid alphaherpesvirus 1 (CHV) belongs to the species *Varicellovirus canidalpha1* within the *Orthoherpesviridae* family, whose genome consists of double-stranded DNA [25]. The latent and subclinical persistence of CHV in dogs poses a significant challenge, potentially leading to infection oversight during routine clinical examination of cAD-MSC donors. Moreover, CHV is distributed globally, with seroprevalence ranging from 21.7% to 80% [26, 27], and nearly one-third of dogs are infected in Croatia [28]. This widespread prevalence makes CHV a considerable risk for contamination during sampling or culturing. Previous studies in human and equine MSCs have shown that herpesvirus infection can decrease the immunomodulatory effects of MSCs [22, 29, 30]. Since studies investigating this phenomenon in canines are currently lacking, this study aimed to assess the susceptibility and adaptability of cAD-MSCs to CHV through serial passages. Moreover, this study sought to explore the impact of CHV infection on the gene expression and secretome composition of cAD-MSCs.

## Methods

### Stem cell culture establishment and characterisation

#### Adipose tissue collection, cAD-MSCs extraction, and propagation

This study obtained adipose tissue samples from 12 clinically healthy dogs (*Canis lupus familiaris*), 11 females and one male who underwent elective surgery. The collection of adipose tissue, extraction of cAD-MSCs and propagation were performed according to previously established protocols [8, 31]. To accomplish the objectives of this investigation, we used cells from cAD-MSC donors 6/21, 9/21, 13/21, 14/21, 1/22, 2/22, 3/22, 6/22, and 7/22, which have been described in a prior publication [8]. In addition to these samples, cells from three novel donors (7/21, 8/21, and 8/22) were used following the same procedure. Nonetheless, this research provides a distinct objective, experimental framework, and conclusions by contrasting the baseline data from uninfected cAD-MSCs with new findings following CHV infection. Table 1 contains information on the age, breed, health status, adipose tissue collection site and mass of the donors. Sterility-tested cAD-MSCs for aerobic and anaerobic bacteria, fungi and mycoplasma were used for all experiments following a previously established protocol [31]. All donor cells were cryobanked in liquid nitrogen via the standard cryobanking procedure with 10% dimethyl sulfoxide (Sigma‒Aldrich, St. Louis, MO, USA, Cat. No. D2650-100ML) at passage 2 (P2) or P3 for future experiments.

**Table 1:** Canine adipose tissue donor information

Donor	Sex	Age (Months)	Breed	Collection site	Surgical procedure	Adipose tissue mass (grams)
6/21*	Female	12	German Spaniel	Ovarian mesostructure	Ovariohysterectomy	7.0
7/21	Female	6	Miniature Schnauzer	Ovarian mesostructure	Ovariectomy	1.4
8/21	Female	12	Mixed	Ovarian mesostructure	Ovariectomy	10.2
9/21*	Female	12	Labrador Retriever	Ovarian mesostructure	Ovariectomy	7.0
13/21*	Female	7	Toy Poodle	Ovarian mesostructure	Ovariectomy	1.0
14/21*	Female	7	Toy Poodle	Ovarian mesostructure	Laparoscopic ovariectomy	1.2
1/22*	Female	10	Jack Russell Terrier	Ovarian mesostructure	Ovariectomy	1.0
2/22*	Female	6	Lagotto Romagnolo	Ovarian mesostructure	Ovariectomy	1.2
3/22*	Female	12	Medium Poodle	Ovarian mesostructure	Ovariectomy	1.0
6/22*	Female	60	Portuguese Water Dog	Ovarian mesostructure	Ovariectomy	2.5
7/22*	Female	36	Mixed	Ovarian mesostructure	Ovariectomy	4.1
8/22	Male	12	German Shepard	Spermatic cord mesostructure	Orchiectomy	2.1

^*^The information from these donors was previously published [8]

#### Immunophenotyping and multipotency testing of cAD-MSCs

As previously described [8], the immunophenotyping and multipotency testing of the cAD-MSCs were performed at P3. FACSVerse (BD, Franklin Lakes, NJ, USA) flow cytometry was used to confirm the immunophenotype, while adipogenic, osteogenic and chondrogenic in vitro differentiation was performed to verify multipotency, following the criteria of the International Society for Cellular Therapy [32].

#### Testing of established cAD-MSCs for CHV

All donors were tested for CHV to gain insight into the possible latent infection of cAD-MSCs extracted from adipose tissue. A cryobanked batch of cells per donor at P2/P3 was first transferred at -20 °C to induce lysis of the cell membranes. After 24 h, the cell lysate was thawed at room temperature for 30 min, vortexed and subjected to nucleic acid extraction using a MagMAX CORE nucleic acid purification kit (Thermo Fisher Scientific, Waltham, MA, USA, Cat. No. A32702) on a KingFisher Flex Purification System (Thermo Fisher Scientific) according to the manufacturer’s instructions. Quantitative real-time PCR (qPCR) was applied for the detection of the CHV glycoprotein B gene according to a previously published protocol [33] using a QuantiFast Pathogen PCR + IC Kit (Qiagen, Hilden, Germany, Cat. No. 211352) on a Rotor-Gene Q (Qiagen) instrument. Beta-actin served as an endogenous control, employing the same reagents, instrument, and a previously established protocol [34]. The reaction mixture setup and thermal cycling conditions were performed as recommended by the manufacturer. The reaction mixtures’ final primer and probe concentrations were adjusted to 1,000 nmol/L for CHV-For (5’-ACAGAGTTGATTGATAGAAGAGGTATG-3’) and CHV-Rev (5’-CTGGTGTATTAAACTTTGAAGGCTTTA-3’) and 500 nmol/L for CHV-Pb (5’-6-FAM-TCTCTGGGGTCTTCATCCTTATCAAATGCG-BHQ1-3’). For beta-actin, primers were adjusted to 83.3 nmol/L for ACT2-1030-F (5’-AGCGCAAGTACTCCGTGTG-3’) and ACT-1135-R (5’-CGGACTCATCGTACTCCTGCTT-3’) and 41.7 nmol/L for ACT-1081-HEX (5’-HEX-TCGCTGTCCACCTTCCAGCAGATGT-BHQ1-3’).

### Isolation and characterisation of autochthonous wild-type CHV

#### CHV recovery from clinical specimen and virus stock production

The autochthonous wild-type CHV strain 29107 was obtained from the organs (liver, spleen, and lungs) of a 6-day-old golden retriever undergoing routine CHV diagnostics at the Croatian Veterinary Institute. The organ samples (1 × 1 cm each) were combined and homogenised with a cold mortar and pestle containing sterile sand and 10 mL of DMEM Low Glucose. The homogenate was freeze-thawed, centrifuged at 2,100 × g for 10 min, filtered using a Millex-HP syringe filter unit 0.45 µm (Merck, Darmstadt, Germany) and stored at − 80 °C. For in vitro propagation, the Madin-Darby Canine Kidney (MDCK) cell line (ATCC, Manassas, VA, USA; Cat. No. CCL-34), which is known to be susceptible to CHV [35], was used. Before inoculation, the MDCK cell line was confirmed to be CHV contamination-free. A 90% confluent MDCK (P34) monolayer in a T25 flask (Thermo Fisher Scientific) was infected with 1 mL of stock supernatant. Following two hours of adsorption at 37 °C (5% CO_2_, 80% humidity), 10 mL of basal medium (79% DMEM Low Glucose (Thermo Fisher Scientific, Cat. No. 31885049), 20% fetal bovine serum (FBS) (Thermo Fisher Scientific, Cat. No. 1027010), and 1% penicillin/streptomycin (Sigma‒Aldrich, Cat. No. P4333-100ML)) was added. Upon full CPE development (monitored using a Lux2 live imaging platform, Axion BioSystems, Atlanta, GA, USA) or 96 h postinfection (p.i.), the infected cell culture flask underwent a single freeze‒thaw-centrifugation cycle. The final CHV stock was generated after the third viral passage on MDCK cells in T75 flasks and stored at -80 °C.

CHV virus stock titration was conducted in triplicate using a confluent MDCK monolayer (P35) seeded in a 96-well microplate (Thermo Fisher Scientific). Eight separate tenfold dilutions of stock supernatant (100 µL per well) were added to the cells. After a two-hour adsorption period, 180 µL of the basal medium was added to the inoculum, and the plates were incubated at 37 °C with 5% CO_2_ and 80% humidity for 72 h. The virus titre (TCID_50_) was calculated using the Spearman–Kärber method.

#### Verification of the autochthonous wild-type CHV strain by NGS

To verify the autochthonous wild-type CHV strain 29107 and generate a whole-genome sequence, we performed next-generation sequencing (NGS). Specifically, viral DNA was extracted from 200 µL of CHV organ suspension homogenate using a DNA Blood and Tissue kit (Qiagen, Cat. No. 69506) according to the manufacturer’s instructions. Sequencing libraries were prepared using the Nextera XT DNA Library Preparation Kit (Illumina Inc., San Diego, USA, Cat. No. 15032354 and No. 15032355) with Nextera DNA UD Indexes (Illumina Inc., Cat. No. 20026934) and sequenced on a NextSeq 550 sequencer (Illumina Inc., Cat. No. SY-415-1002) loaded with a NextSeq 500/550 High Output Kit v 2.5 (300 cycles) (Illumina Inc., Cat. No. 20024908) following the manufacturer’s instructions. Library fragment size control and quantification were performed using a 2100 Bioanalyzer instrument with an Agilent High Sensitivity DNA Kit (Agilent Technologies, Santa Clara, CA, USA, Cat. No. 5067-4626) and a Qubit™ 4 Fluorometer with a Qubit dsDNA HS Assay Kit (Thermo Fisher Scientific, Cat. No. Q32854), respectively.

The sequence reads were assembled into contigs using the Spades software v3.15 [36]. The contigs were compared to known complete CHV genome sequences in NCBI GenBank. Sequence reads were mapped to the GenBank sequence MW353136 using Bwa mem v0.7.17 [37] and SAMtools v1.19 [38] and then used to generate a consensus assembly with Ivar v1.0 [39]. MW353136 was used as a reference because it was the most thoroughly covered by sequencing reads among the complete genome sequences of CHV (taxid:170325) in GenBank. The final novel sequence scaffold of the autochthonous wild-type CHV strain was scaffolded, curated and annotated manually. PROKKA software [40] was used to generate initial functional annotation. The autochthonous wild-type CHV strain 29107 genome sequence was deposited in GenBank under accession number PP349830.

Furthermore, phylogenetic analysis of the novel autochthonous wild-type CHV complete genome sequence was constructed from complete genome alignment of a total of 23 CHV sequences (22 reference sequences from the GenBank database) using IQTree2 software [41] and substitution model HKY + F + I. An optimal substitution model was found using ModelFinder [42]. Multiple sequence alignment was prepared using MAFFT software [43]. The phylogenetic tree was visualised with Python scripting with the help of the module Toytree with UFBoot node support [44] values shown. Calculation of the similarity plot was aided by the Python module Numpy, and visualisation was performed using the Toyplot module. Recombinations were analysed by RDP5 [45].

### In vitro cAD-MSCs infection with wild-type CHV

#### CHV serial passages on cAD-MSCs

To demonstrate successful CHV infection in cultured cAD-MSCs, a cohort of six donors (9/21, 13/21, 14/21, 2/22, 3/22, and 7/22) was randomly chosen for five consecutive viral passage experiments. In contrast to prior experiments involving freshly utilized cAD-MSCs, cryopreserved cAD-MSCs at P2 or P3 were used for these specific infections. After thawing and expansion, cells from each donor were distributed as six replicates into 24-well plates (Thermo Fisher Scientific) at a density of 10^5^ cells/well in 1 mL of basal medium and maintained at 37 °C and 5% CO_2_ (80% humidity) until they reached 90% confluence. Subsequently, the basal medium was removed, and three wells per donor were inoculated with CHV virus stock at a multiplicity of infection (MOI) of 0.5. Following a two-hour incubation period to allow virus adsorption, basal medium was added to the inoculum to a volume of 1 mL. The CHV infection experiments were conducted until the CPE reached 80% or for a maximum of 120 h if the CPE was minimal or absent. Upon meeting the criteria, the plates were frozen at − 80 °C. Thawed cell lysate suspensions from each donor triplicate were individually mixed, transferred to sterile 5 mL tubes (Eppendorf, Hamburg, Germany), and subjected to a second freezing cycle. After the second thaw, the cell lysate suspensions were centrifuged at 2665 × g for 10 min, and the supernatant was filtered through a 0.45 µm filter and stored in 2 mL cryovials (Cryoking, Newcastle, Australia). Subsequent passages of the CHV virus were initiated by preparing new 24-well plates as described previously, with 500 µL/well of the preceding viral passage used as inoculum for virus absorption. Five passages of the CHV virus were conducted for each cAD-MSC donor and control cell line (MDCK). The progression of CPE was documented using a microscopic camera (Axiocam ER/105/208/HD, Axio Observer D1, Zeiss, Jena, Germany) and a Lux 2 live imaging platform. Each passage included three wells of negative controls. Supernatants from each passage (CHV-infected and uninfected) were stored at -80 °C for further qPCR experiments.

#### Quantification of CHV DNA with qPCR

To confirm the success of the infection, i.e., the presence of viral DNA in the supernatant of infected cAD-MSCs, we quantified the number of CHV genome copies. Total DNA was extracted from 200 µL of filtered supernatant from each viral passage after two freeze–thaw cycles using a DNA Blood and Tissue Kit (Qiagen, Cat. No. 69506) according to the manufacturer’s cell extraction protocol. DNA was quantified using a Qubit 1X dsDNA High Sensitivity (HS) kit (Thermo Fisher Scientific, Cat. No. Q33230) on a Qubit 4 Fluorometer (Thermo Fisher Scientific). CHV detection was performed by qPCR as previously described.

For quantification, a triple 5-point standard curve was generated with quantitative genomic DNA from CHV strain D 004 (ATCC VR-552DQ, lot: 70054940). The following values of the standard curve were obtained: R2 = 0.99945, slope = − 3.57263, Y-intercept = 35.24627, and reaction efficiency = 91%. The limit of detection (LOD, ≥ 95% detection in 20 replicates) was 3.31 genomic copies (gc)/reaction. Theoretically, this assay provides an LOD of 6.44 × 10^2^ gc/mL of cell lysate supernatant. The limit of quantification (LOQ, coefficient of variability ≤ 35% in 20 replicates) was set at 136.94 gc/reaction, theoretically providing a CHV LOQ of 2.66 × 10^4^ gc/mL for the cell lysate supernatant. The results are presented as the mean ± SEM unless otherwise stated.

### Gene expression profiling of CHV-infected cAD-MSCs

Gene expression analysis via RT‒qPCR array was conducted on P3 of CHV-infected and uninfected cAD-MSCs from twelve donors. Two T75 flasks (Thermo Fisher Scientific) were seeded with ≈10^6^ cAD-MSCs per flask in basal medium and incubated until they reached 90% confluence (24–48 h). One flask was inoculated with CHV stock at an MOI of 0.5, allowing for a two-hour adsorption period, while the second flask served as a negative control. Twenty-four hours p.i., gene expression profiling was conducted following an established procedure [8]. In brief, RNA extraction was performed with an RNeasy Mini kit (Qiagen, Cat. No. 74106) following the manufacturer’s instructions. The quality of the extracted RNA was verified using an RNA QC kit (Qiagen, Cat. No. 50–727-743). Finally, gene expression profiling was conducted using the RT2 Profiler™ PCR Array for Dog Mesenchymal Stem Cells (PAFD-082ZR, Qiagen). The RNA QC and raw gene expression data from nine uninfected cAD-MSC donors (6/21, 9/21, 13/21, 14/21, 1/22, 2/22, 3/22, 6/22, and 7/22), which were previously published [8], were analysed together with new data from three additional uninfected cAD-MSC donors (7/21, 8/21, and 8/22). This way, a comparative analysis was performed with new results from all 12 CHV-infected cAD-MSC donors.

After data acquisition, the specialised RT2 Profiler PCR Array Data Analysis Software, accessible online at https://dataanalysis2.qiagen.com/pcr (accessed 19 March 2024), enabled normalisation and comprehensive analysis. The gene expression analysis results, researched gene names, symbols, and NCBI sequences are listed in Additional file 1. Statistical significance was determined via Student’s t-test applied to replicated 2^ (-Delta CT) values within both the control and treatment groups with *p* < 0.05. The software automatically established a fold change cut-off value of 2.0, corresponding to a log2fold change ± 1.0. Gene expression profile data were publicly deposited in the NCBI Gene Expression Omnibus database under accession number GSE267402. The data were visualised with GraphPad Prism 10.2.2.

### Proteomic analysis of the CHV-infected cAD-MSCs secretome

The alterations in the proteomic composition of the secretome of cAD-MSCs were also analysed in P3 under two conditions, uninfected and CHV-infected cAD-MSCs, in six randomly selected donors (6/21, 9/21, 14/21, 1/22, 6/22, and 7/22). The cells were seeded in six replicates at 10^5^ cells/mL density in 24-well plates (Thermo Fisher Scientific) and conditioned in the basal medium at 37 °C, 5% CO_2_ and 80% humidity until they reached 90% confluence. The culture medium was then aspirated, and three wells of uninfected cells were rinsed with 2 × 2 mL DMEM Low Glucose before being incubated in 2 mL of the same medium. On the other hand, three wells of CHV-infected cAD-MSCs were inoculated with a MOI 0.5 of CHV viral stock, the virus was allowed to absorb for 2 h, and then the CHV-infected cells were rinsed with 2 × 2 mL of DMEM Low Glucose and incubated in 2 mL of the same medium. Forty-eight hours later, the secretome of the cAD-MSCs was collected as previously described [8].

Following a previously published protocol [8], the samples were prepared for liquid chromatography with tandem mass spectrometry (LC–MS/MS) analysis. In brief, the secretome proteins were reduced and extracted from the culture medium. The protein concentrations were adjusted via the Bradford assay, and enzymatic digestion followed, with peptide separation conducted using the nanoLC EASY-nLC 1200 system (Thermo Fisher Scientific). The mass spectra were recorded using a Q Exactive Plus Hybrid Quadrupole-Orbitrap tandem mass spectrometer (Thermo Fisher Scientific).

Raw data analysis utilised Scaffold Quant Q + S 5.3.0, employing protein sequence data from the *Canis lupus familiaris* reference proteome (UniProt Proteome ID UP000805418, accessed on 30 October 2023, with a total of 20,991 entries). Scaffold Quant version 5.0.3 was utilised for subsequent analysis, employing untargeted label-free quantification and statistical analysis based on spectral counting. Statistical significance, verified via t-tests, was defined as *p* < 0.05, with proteins filtered to include only those with at least two identified peptide sequences. A cut-off value of 1.3 was applied, corresponding to a log2fold change ± 0.3785. The mass spectrometry proteomics data were deposited with the ProteomeXchange consortium via the PRIDE [46] partner repository with the dataset identifiers PXD052289 and 10.6019/PXD052289. In this study, we incorporated previously published raw proteomic data from six uninfected cAD-MSC donors (6/21, 9/21, 14/21, 1/22, 6/22, and 7/22) [8] to facilitate a comparative analysis with new secretome proteome data from six cAD-MSCs following CHV infection.

Bioinformatics analysis of the detected proteins was performed with Gene Ontology (GO) Panther 18.0 to analyse cellular components, protein classes, molecular functions, and biological processes. GO enrichment analysis was used to determine affected protein pathways using Fisher’s exact test and false discovery rate (FDR) correction, with data presented as raw p values < 0.05 and FDR < 0.05. Additionally, a protein–protein interaction network analysis was conducted using STRING (v12.0) [47], employing a high confidence interaction score of 0.700, an FDR < 0.05, and a strength score > 0.75. To elucidate protein pathways and interactions lost due to CHV infection, the proteins secreted distinctively in uninfected samples were grouped with the biologically significant downregulated proteins (uninfected group). In contrast, distinct proteins secreted in CHV-infected samples were grouped with biologically significantly upregulated proteins (CHV-infected group) to elucidate the protein pathways activated after CHV infection. The data visualisation was performed with GraphPad Prism 10.2.2.

## Results

### Extracted cAD-MSCs showed stem cell properties and tested negative for CHV

**Fig. 1:**
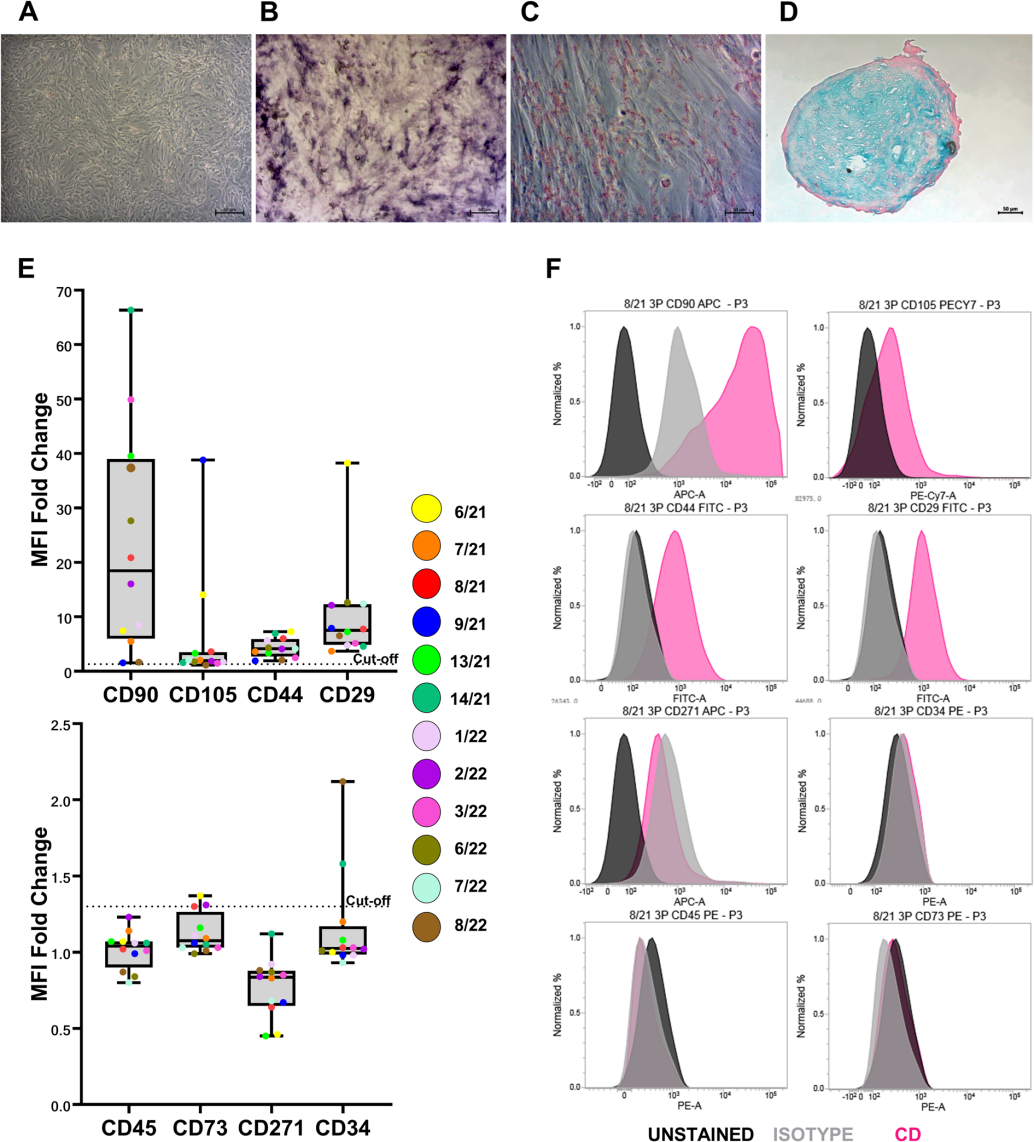
**Canine adipose-derived mesenchymal stem cells (cAD-MSCs) multipotency and stem cell immunophenotype at passage 3 in vitro.** **A** The morphology of undifferentiated cAD-MSCs: **B** osteogenic differentiation marked by the purple staining of alkaline phosphatase activity, **C** adipogenic differentiation evident by the presence of lipid droplets, stained red, **D** chondrogenic differentiation shown by Alcian Blue staining, with aggrecan appearing blue. **A**–**D** Microscopic images were captured using an Axiovert camera on an Axio Observer D1 microscope (Zeiss), with scale bars of 50 μm and 100 μm. **E** Whisker-box plot illustrates the Median Fluorescence Intensity (MFI) fold changes for surface markers CD90, CD105, CD44, and CD29 (upper plot), as well as CD73, CD271, CD45, and CD34 (lower plot) in cAD-MSCs at passage 3. The dotted line indicates the cut-off value of 1.5, and the legend shows individual MFI fold changes for each donor by colourful dots. **F** Representative flow cytometry histograms for each CD marker, with blue representing unstained cells, grey representing isotype controls, and black representing specific CD surface markers. Results of immunophenotype and differentiation analysis from 9 out of 12 canine-adipose derived mesenchymal stem cell donors (6/21, 9/21, 13/21, 14/21, 1/22, 2/22, 3/22, 6/22, 7/22) were previously published [8]

The identity of the stem cells has already been published for nine cAD-MSC donors [8], except for donors 7/21, 8/21 and 8/22. All donors included in this study were positive for the mesenchymal markers CD90, CD105, CD44 and CD29 and negative for the hematopoietic markers CD34 and CD45 (Fig. 1). Furthermore, they differentiated into three mesodermal lineages: adipogenic, as confirmed by the presence of Oil Red O-positive fat droplets; osteogenic, as confirmed by purple staining indicating alkaline phosphatase activity; and chondrogenic, as confirmed by spheroids showing aggrecan (a proteoglycan in articular cartilage) when stained with Alcian blue (Fig. 1).

qPCR analysis revealed that all 12 cAD-MSC donors were negative for CHV.

### The autochthonous wild-type CHV strain was successfully isolated and verified by whole-genome sequencing

**Fig. 2:**
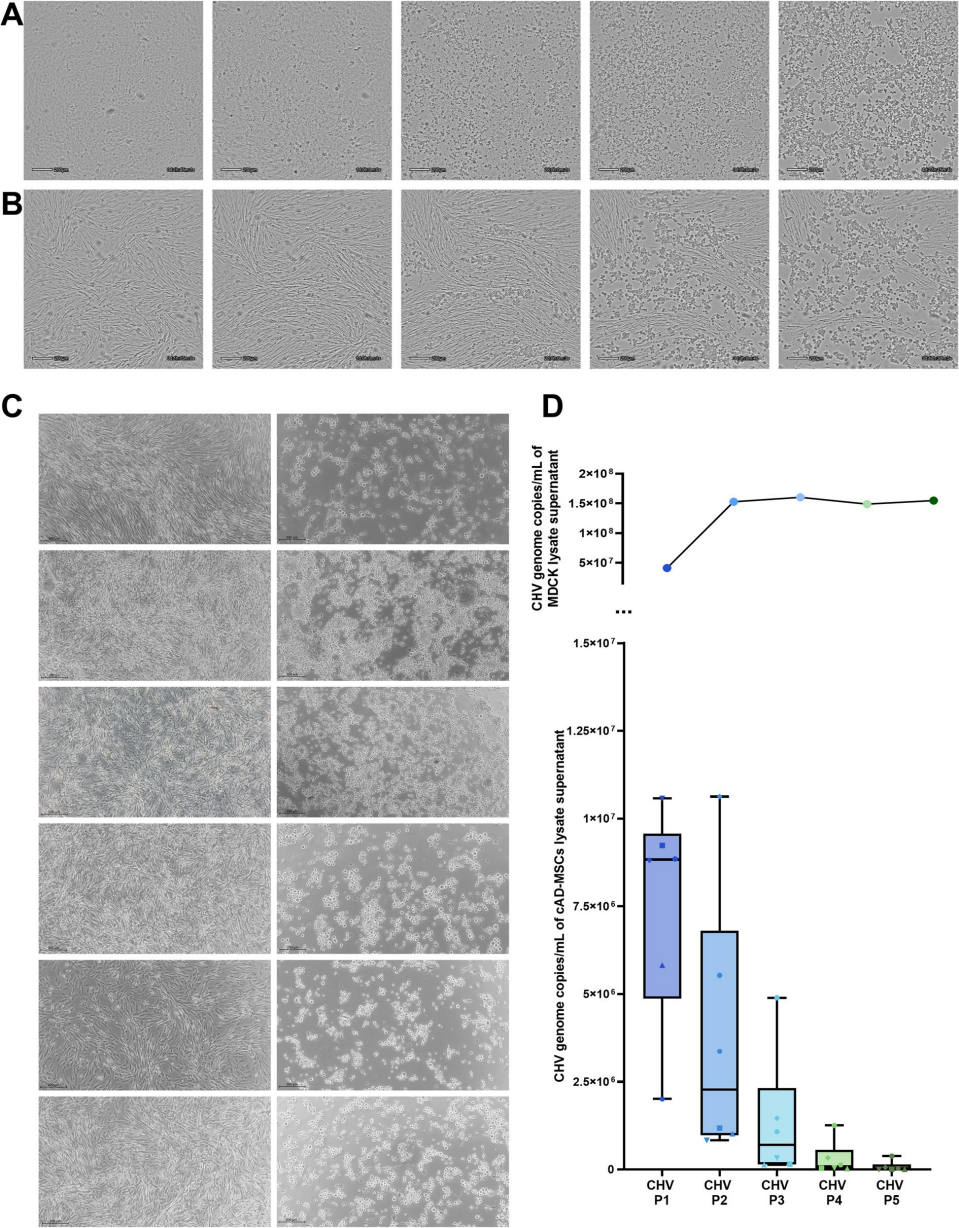
**Development of cytopathogenic effects (CPE) following Canid alphaherpesvirus 1 (CHV) infection in vitro.** **A** Progression of CPE after CHV virus stock infection in the Madin-Darby canine kidney cell line (MDCK) over time (marked in days, hours, minutes, and seconds). **B** Progression of CPE after CHV virus stock infection in canine adipose-derived mesenchymal stem cells (cAD-MSCs) over time (marked in days, hours, minutes, and seconds). **C** cAD-MSC negative control (left) and CPE development in six cAD-MSC donors 48 h after infection with the CHV stock (right). **D** CHV genome copies per mL of cell lysate supernatant for each virus passage in cAD-MSCs (boxplots) and MDCK cells (line graph). Microscopy images were acquired with the Lux 2 live imaging platform (Axion Biosystems) (**A**, **B**) or with the microscopic camera Axiocam ER/105/208/HD on Axio Observer D1 (Zeiss, magnification 50-100x, scale bar 200 µm) (**C**)

The autochthonous CHV strain was successfully recovered from the affected organs and further propagated on the MDCK cell line. The first signs of CPE were observed at ≈30 h p.i., characterised by cell rounding. The number of cells affected by viral activity increased progressively over time. Further progression of CHV infection induced clustering of rounded cells, leaving blank spaces between clusters. The characteristic CPE was fully developed at 96–120 h p.i. (Fig. 2A; Additional file 2). The established virus consistently exhibited CPEs at comparable intervals during the two subsequent passages on MDCK cells used to generate the CHV virus stock. The created CHV virus stock was titrated in triplicate, yielding an average titre of 10^5,04^ TCID_50_/mL.

**Fig. 3:**
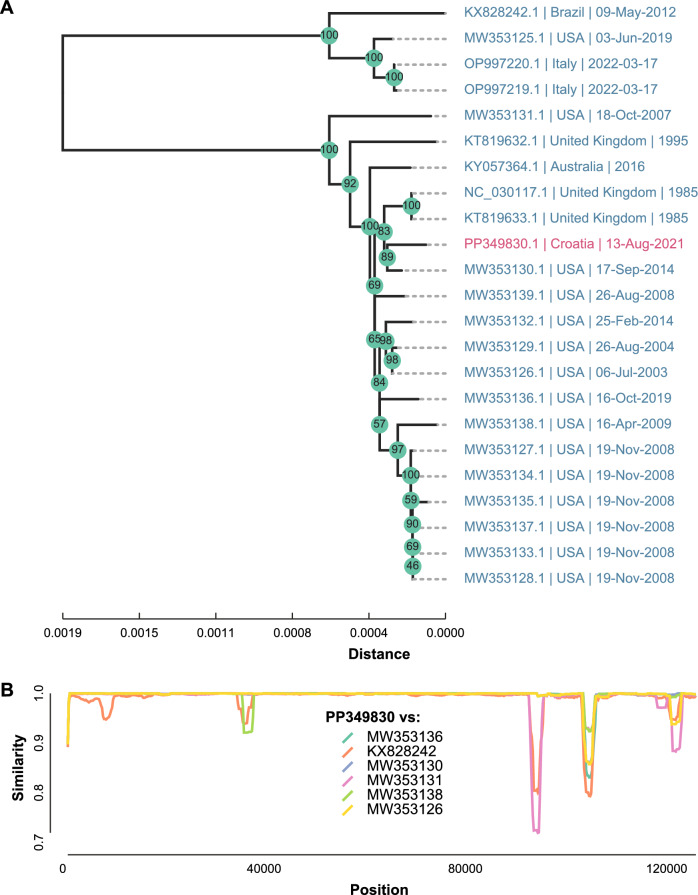
**Phylogenetic analysis of the novel wild-type CHV complete genome sequence.** **A** Phylogenetic placement of the wild-type autochthonous CHV (PP349830) among the 22 complete CHV sequences from the GenBank database. The wild-type CHV is highlighted in pink. Tips are labeled with GenBank accession numbers, collection locations, and dates. **B** Local similarity plot of PP349830 vs representative context sequences (KX828242, MW353130, MW353131, MW353136 and MW353138) along the length of the genome sequence. Context sequences shown were chosen to most representatively sample CHV genome diversity as represented by the phylogenetic tree (**A**). Local hamming similarities versus target sequences were computed in sliding windows (length = 1000 nt, step = 100)

The isolated autochthonous wild-type CHV strain 29,107 was verified by deep sequencing, and the complete genome was characterised. The consensus CHV genome sequence was generated by mapping reads to the GenBank sequence MW353136 and deposited to GenBank under the accession number PP349830. The CHV genome was 124,854 bases long and was covered by 234,338 sequencing reads (150 bp) with a mean base depth of 250 × and a mean base quality of 34.1 (PHRED). Phylogenetic analysis showed that the novel wild-type CHV was most closely related to the GenBank sequence MW353130 (Fig. 3A). MW353130 was most similar to PP349830 along the entire genome length, except in a region spanning positions 102,000 and 106,000, where it was most similar to MW353131 and MW353138 (Fig. 3B). This region encodes one set of copies of the virion proteins US10 and US1 and the regulatory protein V67. The abrupt drop in local similarity to the globally closest sequence suggests possible evolutionary forces, such as recombination, acting on virion proteins under selective pressure. RDP5 analysis detected weak recombination signals in this region of the CHV genome alignment. However, distinguishing recombination from other evolutionary processes is challenging given the similarity levels (global ~ 99%, local 92–93% to MW353131, and 86–87% to MW353130).

The CHV genome sequence contained all 75 alphaherpesvirus genes present in MW353130. The CHV genome contained 41 mutations (18 noncoding mutations, 11 synonymous mutations and 12 nonsynonymous mutations) with respect to MW353130, including seven insertions (four single nucleotides, one dinucleotide and two trinucleotide insertions), eight deletions (four single nucleotides, two dinucleotides, one trinucleotide and one heptanucleotide deletion) and 26 single nucleotide mutations. Coding mutations in CHV were localised to the CHV genes RS1 (n = 6), RS36 (n = 3), US1 (n = 2), and US10 (n = 2) and UL50, UL42, UL37, UL34, UL25, UL8, RL2, US7, US8 and US9 (n = 1 each). Mutations harboured by the wild type CHV are listed in Additional file 3.

### The cAD-MSCs are susceptible to CHV

We demonstrated that CHV can infect cAD-MSCs from all 12 cAD-MSC donors, while six are represented in Fig. 2C. Similar to MDCK cells, all cAD-MSCs displayed focal cell rounding; however, the time to develop CPE was 24–48 h p.i. These rounded cells clustered together, creating empty spaces between them. At 72–96 h p.i., the typical CPE was observed throughout the culture (Fig. 2B; Additional file 4).

However, serial passages of CHV have shown that the time for cAD-MSCs to develop CPE has been prolonged. In CHV P3, CPE was observed in only 2/6 donors, and no observable CPE developed in any of the donor cAD-MSCs in later passages, CHV P4 and CHV P5. The qPCR results corroborated the in vitro observations. The CHV genome copy number decreased with each consecutive passage (Fig. 2D). However, it remained detectable at all five passages, detecting 7,550,146 ± 1,278,419 gc/mL, 3,757,414 ± 1,562,144 gc/mL, 1,338,312 ± 744,417 gc/mL, 301,236 ± 196, 130 gc/mL, and 79,131 ± 60,755 gc/mL in CHV P1, CHV P2, CHV P3, CHV P4, and CHV P5, respectively. Conversely, in the MDCK cell line, the CHV genome copy number increased from P1 to P2 and remained stable across all subsequent passages.

### CHV infection significantly alters the gene expression of cAD-MSCs

**Fig. 4:**
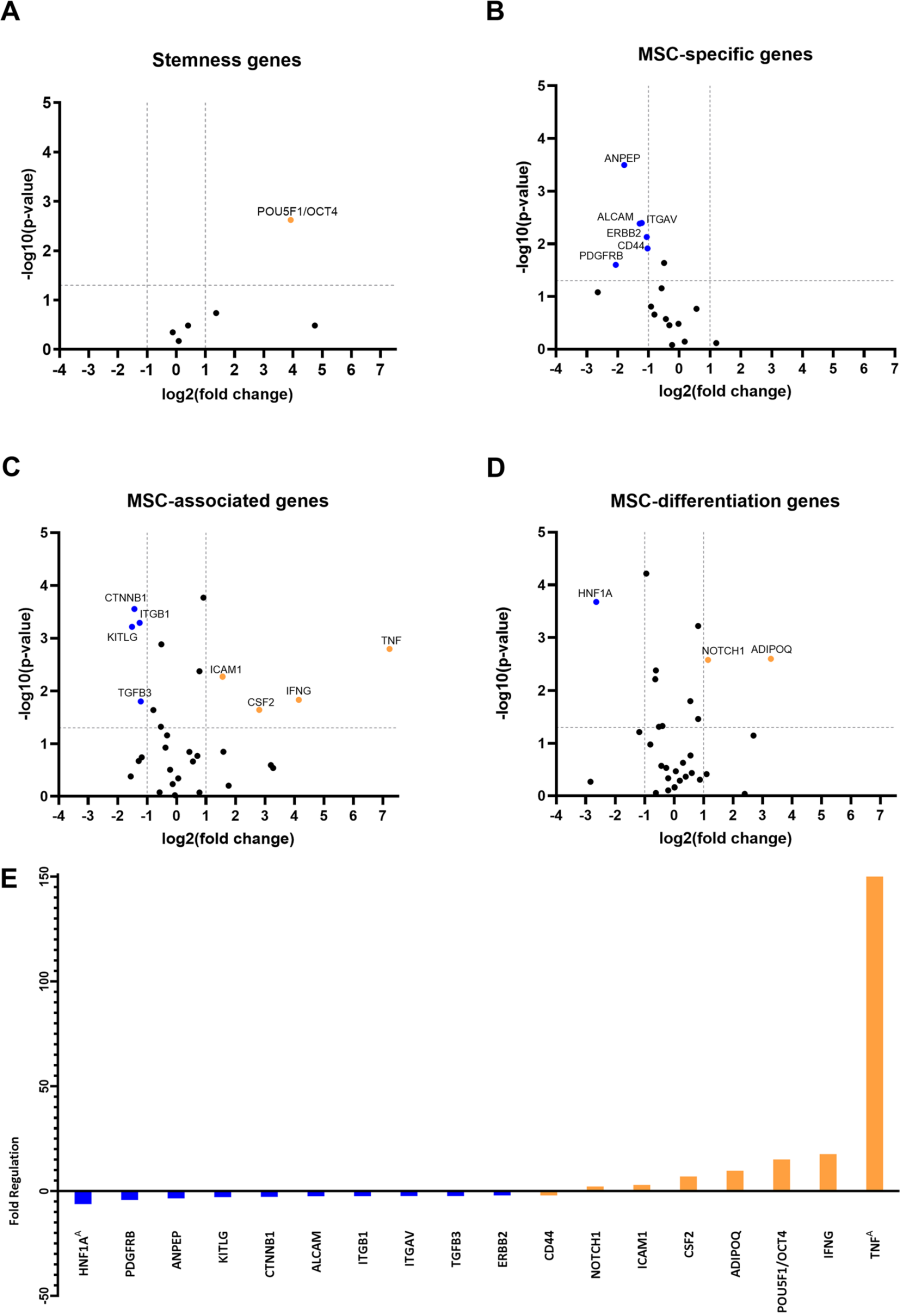
**Gene expression profile of canine adipose-derived mesenchymal stem cells following canine herpesvirus infection in vitro.** Significantly downregulated and upregulated genes are labelled blue and orange, respectively. Volcano plots illustrate the expression profiles of genes related to stemness (**A**), mesenchymal stem cell (MSC)-specific genes (**B**), MSC-associated genes (**C**) and MSC differentiation genes (**D**) after CHV infection. The bar chart shows the fold-regulation of the significantly downregulated and upregulated genes (**E**). ^A^Gene expression was lower in the uninfected sample and more readily detectable in the CHV-infected sample, indicating that the fold change was at least as high as the calculated value

The RNA QC results indicated high RNA quality across all uninfected and CHV-infected samples (Additional file 5). Gene expression analysis revealed significant alterations in 20.9% (18/85) of the total genes included in the array between uninfected and CHV-infected cAD-MSCs. Specifically, expression changes were observed for 16.7% (1/6) of the total stemness genes, 33.3% (6/18) of the total MSC-specific genes, 25.8% (8/31) of the total MSC-associated genes, and 9.7% (3/31) of the total MSC differentiation genes (Figs. 4A–D). Fold regulation values for all downregulated and upregulated genes are shown in Fig. 4E. The full report of the RT2 Profiler PCR Array Data Analysis Software is attached as Additional file 1.

### CHV infection significantly alters protein secretion in the cAD-MSC secretome

**Fig. 5:**
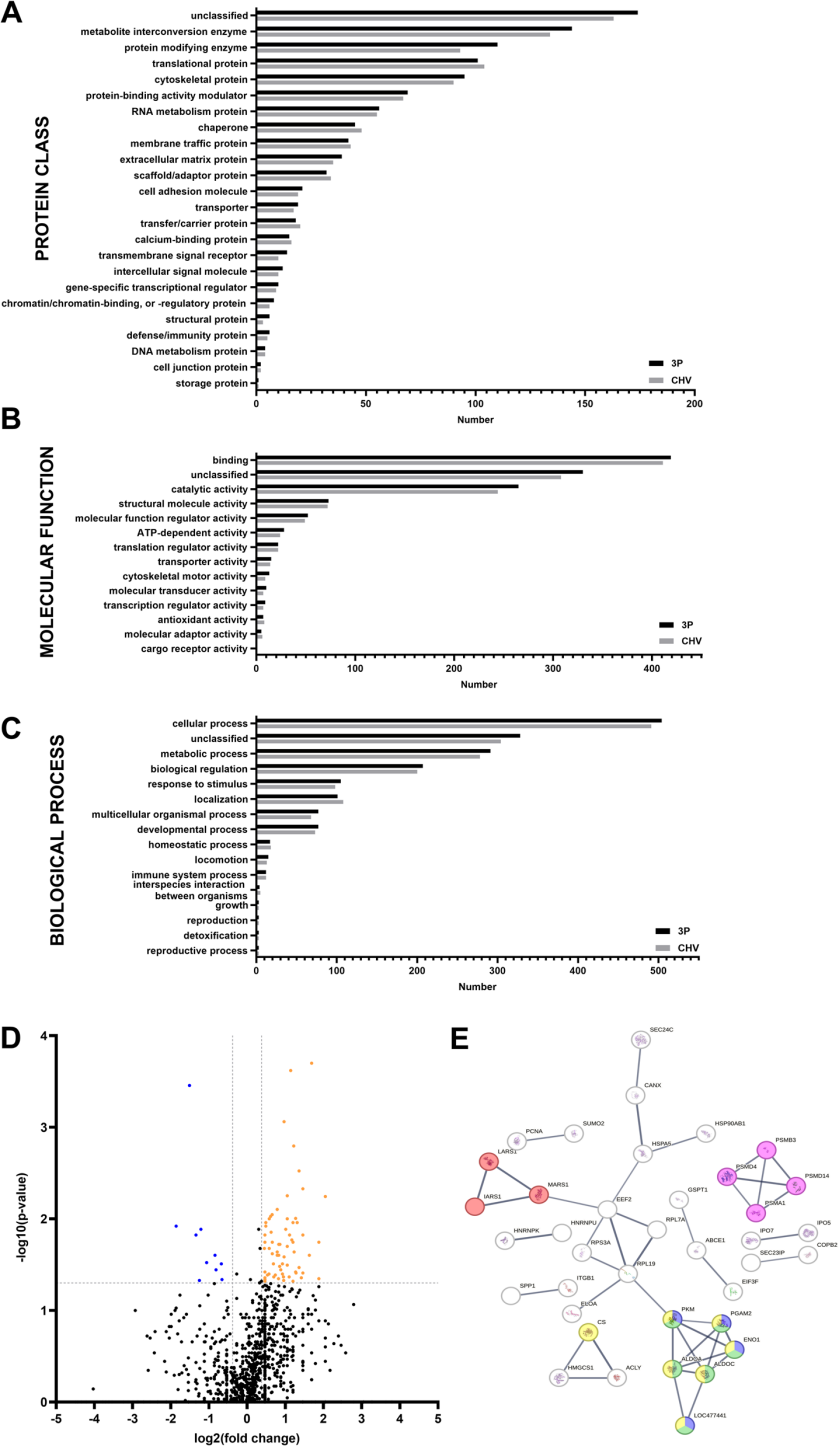
**Proteomic analysis of the secretome from canine adipose-derived mesenchymal stem cells.** **A**–**C** Complete functional protein classification of secretomes comparing uninfected and CHV-infected canine adipose-derived mesenchymal stem cells by protein class (**A**), by molecular function (**B**), and according to biological processes (**C**). Bars indicate the number of categorized secreted proteins. (**D**) Volcano plot showing commonly secreted proteins in the secretome of canine adipose-derived mesenchymal stem cells, comparing uninfected with CHV-infected cells. A comprehensive list of all detected proteins, including their accession numbers, gene names, molecular weights, t-test p values, and fold change data, is provided in Additional File 6. In the graphical representation and Additional file 6, the blue color represents significantly downregulated proteins (p < 0.05) with a fold change exceeding 1.3. Conversely, the orange color signifies proteins demonstrating statistical significance (p < 0.05) in upregulation, with a fold change greater than 1.3. (**E**) String protein interaction network representing significant protein interactions occurring due to CHV infection. The coloured nodes represent interactions with a high confidence score (0.700) and strength > 0.75. Red represents interactions of proteins involved in the aminoacyl-tRNA synthetase multienzyme complex; pink represents interactions involving the proteasome complex; yellow represents interactions involving pyruvate metabolism and carbon metabolism; green represents interactions involving glycolysis; and blue represents interactions involving the enolase, C-terminal TIM barrel domain and glycolysis

The proteomic analysis of the cAD-MSC secretome identified 1,181 proteins. A comprehensive list of all detected proteins, including their accession numbers, gene names, molecular weights, t-test p values, and fold change data, is provided in Additional File 6. The 86.8% (1,025/1,181) of proteins were common between uninfected and CHV-infected samples. The commonly detected proteins were further compared by GO enrichment analysis, i.e., cellular components, protein classes, molecular functions, and biological processes (Fig. 5A–C). Similar involvement in all annotated functions was observed. Analysis of cellular components revealed that ≈ 60% (788/1181 and 754/1181 in uninfected and CHV-infected samples, respectively) of proteins belonged to cytoskeletal proteins, ≈ 19% (239 in uninfected and 235 in CHV-infected samples) belonged to protein-containing complexes, and the rest (154 in uninfected and 192 in CHV-infected) were not assignable to cellular component GO terms. More than 50% of the cAD-MSC secretome proteins were metabolite conversion enzymes, translational proteins, protein-modifying enzymes, and cytoskeletal proteins (Fig. 5A). Their molecular functions were mainly binding and catalytic activity (Fig. 5B), and they were predominantly involved in cellular and metabolic processes, biological regulation, response to stimuli and localisation (Fig. 5C). Among the commonly secreted proteins, 10 were significantly downregulated, whereas 66 were significantly upregulated (Fig. 5D; Additional file 6).

Within the subset of proteins exhibiting distinctive secretion patterns (comprising 13.2% of the total proteins), 105 proteins were specific to uninfected samples, whereas 51 proteins were specific to CHV-infected samples. Subsequent bioinformatic analysis was subsequently conducted to gain insight into protein pathway involvement. The results of the GO enrichment analysis are presented in Table 2, while the outcomes of the STRING analysis are detailed in Table 3 and Fig. 5F.

**Table 2:** Protein pathway analysis of the uninfected and CHV-infected canine adipose MSC secretomes via Gene Ontology Panther

	Gene Ontology Panther protein pathways analysis	Raw *p* value*	False discovery rate (FDR)*	Gene names of proteins involved in the pathway
Uninfected group	Nicotinic acetylcholine receptor signalling pathway	2.09E-04	3.37E-02	MYH1, MYH2, MYH4
	Cytoskeletal regulation by Rho GTPase	2.09E-04	1.69E-02	MYH1, MYH2, MYH4
	Wnt signalling pathway	7.28E-04	3.90E-02	CDH13, CDH2, MYH1, MYH2, MYH4
CHV-infected group	Pyruvate metabolism	6.42E-05	3.45E-03	ACLY, CS, PKM
	Cell cycle	4.89E-04	1.97E-02	PSMD4, EIF3F, PSMD14
	Glycolysis	6.92E-06	5.57E-04	ALDOA, ALDOC, ENO1, PGAM2, LOC477441

*Analysis criteria were raw p values < 0.05 and FDR < 0.05

**Table 3:** Protein pathway analysis of the secretome of uninfected and CHV-infected canine adipose MSCs via STRING

	STRING protein interaction analysis	Strength score*	False discovery rate*	Gene names of proteins involved in interaction
Uninfected group	Extracellular matrix organisation, and Extracellular matrix	1.24	5.58E-08	ECM2, POSTN, ADAMTS1, FBLN5, MFAP2, BGN, MMP12, MMP9, PCOLCE2, COL6A1, PRELP, ITGBL1
	Extracellular matrix organisation, and Extracellular matrix	1.22	3.25E-07	ECM2, POSTN, ADAMTS1, FBLN5, MFAP2, BGN, MMP12, MMP9, PCOLCE2, COL6A1, PRELP
	Mixed, incl. Collagen formation, and Degradation of the extracellular matrix	1.19	1.79E-05	POSTN, ADAMTS1, FBLN5, MFAP2, BGN, MMP12, MMP9, PCOLCE2, COL6A1
	Collagen formation, and Molecules associated with elastic fibres	1.27	2.32E-05	POSTN, ADAMTS1, FBLN5, MFAP2, BGN, MMP12, PCOLCE2, COL6A1
	Collagen biosynthesis and modifying enzymes, and Proline metabolic process	1.3	8.20E-04	POSTN, ADAMTS1, BGN, MMP12, PCOLCE2, COL6A1
	Collagen biosynthesis and modifying enzymes, and Fibrillar collagen, C-terminal	1.39	2.40E-03	POSTN, BGN, MMP12, PCOLCE2, COL6A1
	Mixed, incl. Glycosaminoglycan degradation, and Glycosphingolipid metabolism	1.28	6.50E-03	CLN5, TPP1, NAGLU, MAN2B1, GLB1
	Mixed, incl. Exopeptidase activity, and Maturity onset diabetes of the young	1.07	8.50E-03	LOC102156614 (CTSL), PAM, LGMN, CTSK, DPP7, CPE
	Collagen biosynthesis and modifying enzymes, and Fibrillar collagen, C-terminal	1.36	1.92E-02	POSTN, BGN, MMP12, COL6A1
	Mixed, incl. Banded collagen fibril, and small leucine-rich proteoglycan, class I, decorin/asporin/byglycan	1.61	3.57E-02	POSTN, BGN, COL6A1
	Mixed, incl. Exopeptidase activity, and Papain family cysteine protease	1.22	4.88E-02	LOC102156614 (CTSL), LGMN, CTSK, DPP7
	Mixed, incl. Glycosaminoglycan degradation, and Glycosphingolipid metabolism	1.22	4.88E-02	CLN5, TPP1, NAGLU, GLB1
CHV-infected group	Glycolysis	1.49	3.90E-04	ENO1, ALDOA, ALDOC, LOC477441, PGAM2, PKM
	Enolase, C-terminal TIM barrel domain, and Glycolysis	1.73	2.30E-03	ENO1, LOC477441, PGAM2, PKM
	aminoacyl-tRNA synthetase multienzyme complex	2.08	3.80E-03	MARS1, IARS1, LARS1
	Pyruvate metabolic process, and Carbon metabolism	0.97	6.50E-03	ENO1, ALDOA, ALDOC, LOC477441, CS, PGAM2, PKM
	Proteasome complex	1.24	3.49E-02	PSMD14, PSMD4, PSMB3, PSMA1

*The analysis criteria were a high confidence interaction score of 0.700, FDR < 0.05, and strength score > 0.75

## Discussion

In vitro manipulations are unavoidable for acquiring and conserving therapeutic quantities of cAD-MSCs and their secretome. Nevertheless, these procedures involve an inherent risk of microbial contamination and the potential spread of pathogens, including viruses, originating from infected donor cells. Therefore, we aimed to investigate the interaction between globally distributed CHV and cAD-MSCs and to assess whether CHV infection affects the gene expression and secretome composition of cAD-MSCs. To the best of our knowledge, this study represents the first investigation of the effects of viral infection on the gene expression and secretome profile of cAD-MSCs. To achieve the aim, an autochthonous CHV strain was established. This strain should reflect the natural virus-host interplay more accurately than culture-adapted and extensively propagated CHV strains in vitro. The novel complete genome sequence of the autochthonous CHV strain from Croatia contributes to the knowledge of the complete genome diversity of CHV. Prior to this contribution, the GenBank database contained 22 complete CHV genome sequences, with only five sequences from Europe, three from the United Kingdom and two from Italy.

In vitro susceptibility to CHV, as indicated by the characteristic CPE of *Orthoherpesviridae* viruses [48], was likewise observed in cAD-MSCs and MDCK cells (Fig. 2A, B; Additional files 2 and 4). However, successive passages of CHV on cAD-MSCs exhibited a gradual reduction and disappearance of CPE, which was corroborated by diminishing yet persistent CHV genome copy numbers (Fig. 2C) in the supernatants of cell lysates, indicating abortive infection. The abortive infection has recently been documented in herpes simplex virus in vitro research [49], which proves that herpesviruses can infect nonneuronal cells, remain quiescent and be reactivated, challenging the current paradigm of herpesvirus latency. Our results seem to resemble the above scenario, but further experimental validation is needed to assign abortive infection status to this specific virus-host interaction.

The effects of CHV infection on cAD-MSCs were further explored at the gene expression level, and the results revealed that CHV infection significantly affected the expression of researched genes (Fig. 4). The upregulated genes were associated with proliferation [50], differentiation [51], and the immunosuppressive response [52–54]. Similar alterations in gene expression attributed to virus infections have been documented in previous studies on human stem cells [21, 55]. The most significantly upregulated gene in infected cAD-MSCs, *TNF* (Fig. 4C), encodes a protein responsible for various cellular processes, including proliferation, differentiation, and, interestingly, immune suppression in MSCs [54, 56]. It remains unclear whether canine MSCs can produce TNF; however, in human MSCs, there is clear evidence of their inability to produce TNF [57]. Nevertheless, cAD-MSCs are adipocyte progenitors, and adipocytes and their progenitors are well known for secreting TNF [58]. In this study, high *TNF* production combined with alterations in other genes primarily suggested increased adipocyte differentiation in CHV-infected cells. Moreover, the upregulation of *ADIPOQ* and *NOTCH1* [59, 60] coupled with the downregulation of several genes related to stemness and regenerative capacity [61–64] further support the initiation of differentiation processes, predominantly adipogenesis, in CHV-infected cAD-MSCs. These findings indicate that CHV infection may drive cAD-MSC differentiation, affecting their regenerative potential and altering their typical stem cell properties.

The proteomic composition of the cAD-MSC secretomes further corroborated the initiation of adipogenesis in CHV-infected cells observed at the RNA level. GO enrichment analysis of the uninfected group revealed that proteins significantly involved in the WNT signalling pathway were absent in CHV-infected cells (Table 2). This finding suggests that CHV infection leads to the loss of the WNT signalling pathway, and its deactivation in MSCs is considered crucial for inducing adipogenesis [65, 66]. Furthermore, both GO enrichment and STRING analyses (Tables 2 and 3) revealed the presence of essential protein pathways involved in cell self-renewal, structure, survival, homing, and migration [67, 68] in the uninfected group, which were lost after CHV infection. These losses at the proteomic level align with the microscopically observed loss of cellular structure, survival, and migration following CPE development (Fig. 2; Additional files 2 and 4).

Additionally, the observed microscopic reduction in cell survival was further supported by findings from the CHV-infected group cAD-MSC secretomes. GO enrichment and STRING analyses revealed upregulated glycolysis and elevated levels of proteins associated with the enolase and pyruvate metabolism pathways (Tables 2 and 3). This virus’s takeover of host cell resources and metabolic machinery prioritises viral particle production over normal cellular functions, ultimately leading to cell damage and death. Similar alterations were previously documented in studies on viral-host interactions in other *Orthoherpesviridae* infections, such as human cytomegalovirus [69] and herpes simplex virus [70, 71] infections.

Although novel knowledge is unravelled in this study, future studies should deepen the understanding of cAD-MSC-CHV interactions by examining transcriptome and secretome alterations throughout serial passages and identifying any coinciding variants in the viral genome. Furthermore, as the AD-MSC secretome has recently been shown to have an antiviral effect [72] in felines, our research provides a direction for overcoming the currently limited knowledge on utilising stem cells to treat viral diseases in canines.

In conclusion, our study demonstrated the susceptibility of cAD-MSCs to CHV infection. The observed genomic variations in gene expression indicate potential impacts on the stemness, migration, and other functional properties of cAD-MSCs, highlighting the need for further studies to evaluate their functional capacity post-infection. Moreover, gene expression and secretome analyses suggest a shift in stem cell differentiation toward an adipogenic phenotype. These cumulative changes can negatively impact the regenerative properties of cAD-MSCs. These findings highlight the critical importance of screening cAD-MSC batches intended for therapeutic applications to ensure the absence of CHV prior to administration.

## Supplementary Information

Additional file 1 This additional report was generated through the analysis of gene expression data employing the RT² Profiler PCR Array Data Analysis Software. In addition to the analysis results, the investigated gene names, symbols, and corresponding NCBI sequences were comprehensively described. (PDF 321 KB)

Additional file 2 This movie additionally illustrates the progression of cytopathogenic effects in the Madin-Darby canine kidney cell line after infection with canid alphaherpesvirus 1. A scale bar of 200 µm is provided, along with a time bar delineated in days, hours, minutes, and seconds. (MP4 18067 KB)

Additional file 3 The table lists all mutations found in the wild-type CHV (GenBank accession number PP349830) relative to the closest relative, MW353130. Mutation positions, effects, gene genes affected, and protein-level consequences are also listed. (XLSX 13 KB)

Additional file 4 This movie additionally illustrates the progression of cytopathogenic effects in canine adipose-derived mesenchymal stem cells after infection with canid alphaherpesvirus 1. A scale bar of 200 µm is provided, along with a time bar delineated in days, hours, minutes, and seconds. (MP4 20489 KB)

Additional file 5 This additional table displays the RNA quality control (QC) analysis results, including the RNA integrity score and 28:18S ratio for all assessed total RNA samples. (XLSX 10 KB)

Additional file 6 This table provides each identified protein’s accession number, gene name, and molecular weight. Furthermore, t-tests were used to determine the significance and fold changes in secretome protein secretion between CHV-infected and uninfected samples. The list is organised in ascending order based on the t-test p values. The blue color represents significantly downregulated proteins (p < 0.05) with a fold change exceeding 1.3. Conversely, the orange color signifies proteins demonstrating statistical significance (p < 0.05) in upregulation, with a fold change greater than 1.3. A “missing value” indicates that the protein is secreted exclusively in uninfected samples, while a “reference missing” indicates that the protein is secreted solely in CHV-infected samples. (XLSX 108 KB)

## Data Availability

The datasets generated and analysed during the current study are available in the NCBI GenBank [https://www.ncbi.nlm.nih.gov/nuccore/PP349830], Gene Expression Omnibus repository [https://www.ncbi.nlm.nih.gov/geo/query/acc.cgi?acc=GSE267402] and ProteomeXchange repository [https://www.ebi.ac.uk/pride/archive/projects/PXD052289]. This manuscript and additional files include other data generated or analysed during this study.

## Abbreviations

cAD-MSCs: Canine adipose-derived mesenchymal stem cells
CD: Cluster of differentiation
CHV: Canid alphaherpesvirus 1
CPE: Cytopathogenic effect
DMEM Low Glucose: Dulbecco’s modified eagle medium with low glucose
FBS: Fetal bovine serum
FDR: False discovery rate
GO: Gene ontology
LOD: Limit of detection
LOQ: Limit of quantification
MDCK: Madin-Darby canine kidney cell culture
MSC: Mesenchymal stem cell
NGS: Next-generation sequencing
P(number): Passage (number)
p.i.: Postinfection
qPCR: Quantitative polymerase chain reaction
RT-qPCR: Reverse transcription-quantitative polymerase chain reaction
TCID50: 50% tissue culture infectious dose.
